# The face of appearance-related social pressure: gender, age and body mass variations in peer and parental pressure during adolescence

**DOI:** 10.1186/1753-2000-7-16

**Published:** 2013-05-17

**Authors:** Susanne Helfert, Petra Warschburger

**Affiliations:** 1 Department of Psychology, University of Potsdam, Karl-Liebknecht-Str. 24/25, 14476, Potsdam, OT Golm, Germany

**Keywords:** Peer pressure, Parental pressure, Adolescence, Gender, Age, BMI

## Abstract

**Background:**

Appearance-related social pressure plays an important role in the development of a negative body image and self-esteem as well as severe mental disorders during adolescence (e.g. eating disorders, depression). Identifying who is particularly affected by social pressure can improve targeted prevention and intervention, but findings have either been lacking or controversial. Thus the aim of this study is to provide a detailed picture of gender, weight, and age-related variations in the perception of appearance-related social pressure by peers and parents.

**Methods:**

1112 German students between grades 7 and 9 (mean age: M = 13.38, SD = .81) filled in the Appearance-Related Social Pressure Questionnaire (German: FASD), which considers different sources (peers, parents) as well as various kinds of social pressure (e.g. teasing, modeling, encouragement).

**Results:**

Girls were more affected by peer pressure, while gender differences in parental pressure seemed negligible. Main effects of grade-level suggested a particular increase in indirect peer pressure (e.g. appearance-related school and class norms) from early to middle adolescence. Boys and girls with higher BMI were particularly affected by peer teasing and exclusion as well as by parental encouragement to control weight and shape.

**Conclusion:**

The results suggest that preventive efforts targeting body concerns and disordered eating should bring up the topic of appearance pressure in a school-based context and should strengthen those adolescents who are particularly at risk - in our study, girls and adolescents with higher weight status. Early adolescence and school transition appear to be crucial periods for these efforts. Moreover, the comprehensive assessment of appearance-related social pressure appears to be a fruitful way to further explore social risk-factors in the development of a negative body image.

## 

Factors influencing the development of a negative body image during adolescence have received increasing attention due to the fact that body dissatisfaction is highly prevalent among adolescents in western society and is also one of the main predictors of low self-esteem, depression, and not least of all disordered eating [1-3]. Sociocultural influences are particularly relevant in this process. Thompson’s Tripartite Influence Model [4] of body dissatisfaction and Stice’s Sociocultural Model of Disordered Eating [5] have identified media, peers, and parents as the three formative sociocultural influences. Many studies have emphasized the crucial role of perceived appearance-related social pressure in the development of body dissatisfaction and disordered eating. Thus, social agents – especially peers and parents, who are closest to the adolescent – both consciously and unconsciously convey and enhance appearance-related norms through direct and indirect interactions [5,6]. Peers and parents promote the construction of beauty ideals, norms, and standards and highlight the importance of appearance. Numerous studies have investigated different aspects of peer [e.g. [1,7-9] and parental pressure [e.g. [10-16]. However, to our knowledge no theoretical framework has yet integrated the main influences from both peers and parents discussed in the literature. In order to develop a comprehensive measure of appearance-related pressure from peers and parents (see [17]), we reviewed the literature and found influences from *friends*[1,2] and *schoolmates* as well as *teasing* or *exclusion* to be the most established peer influences. With regard to parental influences, aspects such as *parental norms and modeling* behavior regarding appearance [e.g.[10-12], parental disregard or *ignorance* [e.g.[13] as well as *teasing* [e.g.[9,14] and *encouragement to control weight and shape* [e.g.[13,15,16] have been found to affect the body image of adolescents (see Figure 1).

**Figure 1:**
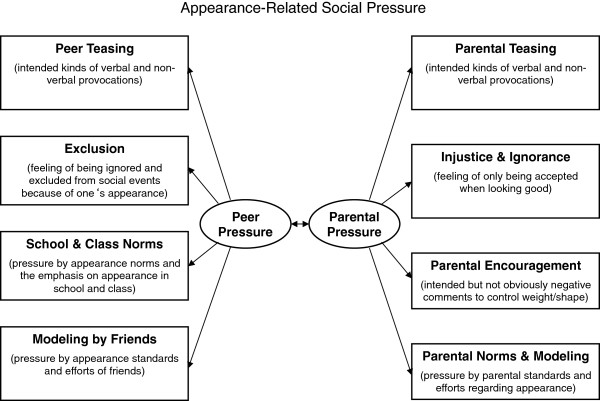
Considered aspects of appearance-related social pressure.

Up to now, research has provided important findings on the impact of single types of social pressure and general behavioral mechanisms. However, in order to explain the development of negative body image and design targeted prevention approaches, we must also find out who is particularly faced with social pressure. The following sections will attempt to summarize the knowledge on variations according to individual characteristics considering gender-, age, and weight-related variations.

## Gender variations

Because studies on social pressure have mostly derived from eating disorder and body image research, they have often concentrated on girls, for whom they reported a higher amount of appearance-related *influences from friends* [e.g. [16,18], more fear of *exclusion* by peers because of one’s appearance [19] and a greater importance of *school and class norms*[20]. These findings appear quite plausible with regard to the particular emphasis placed on female beauty and appearance in western society. However, during the last ten years research has also considered boys and revealed that some of the gender differences might be due to inadequate instruments for boys (i.e., only focusing on the thin ideal [21,22]). Consequently, studies that used measures without that bias suggested comparable processes of appearance-related interactions with friends and social exclusion for both girls and boys [7,23].

Findings regarding gender differences involving parental pressure have been sparser but therefore less controversial. They predominantly support the conclusion that appearance is more heavily emphasized among girls. Consequently, girls perceived a greater extent of *parental appearance norms and modeling* behavior *(e.g.,* parental concerns with body shape, efforts to look good [6,16,24]). Interestingly, studies investigating *parental encouragement to control weight and shape* found no gender difference [13,16,25]. However, this might be due to the focus on encouragement to diet, which might be used by parents regardless of their child’s gender when the child is at risk of becoming overweight. We suppose that if an operationalization of “encouragement” without the bias towards the thin ideal is applied, gender differences might occur. Concerning parental disregard (i.e., *injustice and ignorance*) studies are rare. The study of Meesters et al. [13] among Dutch adolescents aged 10 to 16 provided important suggestions regarding the influential role of parental rejection or insecure attachment in the development of body concerns but could not find gender variations. However, this aspect of parental pressure requires further investigation.

Findings on *peer and parental teasing* have been particularly inconsistent. While in some studies [26] girls were more frequently faced with peer teasing, others did not find any gender difference [18,27] or even found more teasing experiences among boys [7,16]. The same applies with parental teasing*.* Some studies did not find a gender difference [6,16] and others have revealed that girls perceive more parental teasing [24,27]. These inconsistencies might result from the measurement of teasing as isolated indices or as combinations of peer and parental teasing. Hence, validity and reliability might have been restricted.

## Age-related variations

Developmental theories on the transformation of relationships with peers and parents [28] suggest that social pressure might change throughout adolescence. Further, the gender intensification hypothesis of Hill and Lynch [29] suggests that pressure from peers and parents to conform to gender roles, behavior and appearance standards intensifies during adolescence. However, only a few studies have investigated developmental effects in the field of social pressure and reported a growing *influence of friends* and an increase in appearance pressure by other peers (e.g., schoolmates) during middle adolescence [1,7]. In addition, Dohnt and Tiggemann [30] provided interesting findings on the impact of *school and class norms* among elementary school girls in the first four years of formal schooling. While girls in the first year at school thought that their peers would desire a larger figure, girls from grade two to four already assumed that their peers desired a thinner figure. These results suggest that orientation towards a certain body ideal as well as appearance-related school and class norms develop very early. Interestingly, Chen and Jackson [31] reported an age-gender interaction among a sample of Chinese adolescents, suggesting that appearance conversations between friends might increase with age only among girls but not among boys. However, they could not establish a comparable effect regarding general appearance-related pressure. In contrast to a probable increase in appearance-related interactions, *teasing* and *exclusion* proved to be rather stable during adolescence [7]. Jones [1] even found a decrease in reported teasing among adolescents from grades 10 to 11, which indicates that teasing becomes less important with the transition to adulthood.

To our knowledge, no study exists that considered age-related variations in *parental pressure,* but developmental theories have suggested a decrease in adult orientation and an increase in peer orientation for appearance-related issues beginning in early adolescence [28,32,33]. This might lead to the conclusion that parental pressure has either a stable or even a shrinking relevance during adolescence. However, Striegel-Moore and Kearney-Cooke [34] revealed that American parents become more critical of their children’s physical attractiveness as the children grow older. Hence, appearance-related pressure (e.g., *encouragement to control weight and shape*) might also increase.

However, because findings on parental pressure have been incomplete and knowledge of age-related trends in peer pressure comes from a few predominantly cross-sectional studies, we should be cautious about drawing conclusions for age-related trends.

## Body mass variations

Many studies have examined stigmatization of overweight and obese persons. As appearance stigmatization is a distinct and serious form of social pressure, including *peer teasing* and *exclusion* alike, it can be concluded that overweight persons per se experience more of these kinds of pressure [9]. Beyond that, a few studies have also suggested a higher amount of teasing experiences among underweight adolescents [26]. The results of Jones and Crawford [7] even suggest an interaction of weight and gender: While particularly overweight girls experienced teasing and fear of exclusion, underweight boys displayed the highest scores. These results were interpreted with regard to the different beauty ideals for men and women: Girls who do not fit the slim norm and boys who do not fit the bulky, muscular male ideal are more exposed to stigmatization. However, the findings have left the question unanswered whether deviating from normal weight per se increases the risk of being subjected to more direct peer pressure or whether weight-related variations are different for girls and boys.

To our knowledge, only Jones and Crawford [7] have considered weight variations in more subtle forms of peer pressure and found that adolescents with higher BMI perceived stronger *influences from friends* and general appearance pressure by peers (e.g., *schoolmates*).

Studies reporting relationships between weight status and parental pressure are even sparser. A few studies reported higher scores in *parental teasing* among overweight boys and girls [24,26,35]. Regarding *parental encouragement* to lose weight, Wertheim et al. [25] found a moderate positive association with weight status for early adolescent boys and girls alike. Unfortunately, the study did not consider muscle gaining. Finally, Rodgers et al. [24] could not find an association between weight status and the perception of appearance-related *parental norms and modeling* behavior.

In summary, more knowledge on variation according to individual characteristics is needed to explain the development of negative body image and to design targeted prevention approaches. While previous studies have provided important findings on the impact of single types of social pressure and general behavioral mechanisms, findings on gender, age and weight variations in different aspects of social pressure have either been incomplete or controversial, because only a few studies have explicitly focused on these individual differences. Moreover, due to restricted sample size most of the studies could not consider possible interactions between the three factors. Finally, research has often concentrated on girls, or when it included boys, the applied measures often contained a bias towards the thin ideal that is not suitable for boys.

Thus, research still remains limited for the purpose of drawing firm conclusions about gender, body mass variations and age-related trends in the perception of social pressure.

## Hypotheses

The current study attempts to contribute to an enhancement of current theories on appearance-related social pressure by investigating the occurrence of different types of pressure in a large sample of German adolescent boys and girls. Moreover it provides a comprehensive exploration of differential effects of gender, weight, and grade as well as interactions among these factors. Based on previous findings, we expected the following:

## Gender variations

1. The research of the recent years has posed the question whether the emphasis placed on female beauty sets girls at greater risk for appearance-related social pressure or whether these effects have derived from biased instruments that were unsuitable for boys. Even if several studies have pointed to the growing relevance of appearance among boys and some gender differences diminished when studies use muscle- and weight-related instruments, most of the findings suggest that the focus on appearance is still stronger for females. Consequently, we hypothesized that girls would show higher levels of peer pressure through *modeling by friends*, *school and class norms*, *peer teasing* and *exclusion* as well as higher levels of parental pressure through *parental teasing*, *encouragement to control weight and shape*, *parental norms and modeling* and *injustice and ignorance*.

## Grade-level variations

2. Previous findings have brought evidence for an age-related increase of appearance orientation and modeling processes among adolescents whereas more direct aspects of peer pressure have proven to be quite stable. We thus hypothesized that *modeling by friends* and perceived *school and class norms* would be higher in older compared to younger adolescents. To take account of the findings of Chen and Jackson [31] we also want to test for an interaction between age and gender.

3. Regarding parental pressure, findings are rare and therefore we based our expectations on developmental theories. These theories have suggested that parents are not the main source of appearance-related standards and thus parental norms and modeling should not differ by grade. However, parents have been found to become more concerned with the physical attractiveness of their adolescent child. Thus, we expected that *parental encouragement to control weight and shape* would be more prevalent among older adolescents.

## Body mass variations

4. Finally, research has raised the hypothesis that either a) higher weight status per se sets individuals at greater risk for stigmatization or b) girls with higher weight are stigmatized if they do not fit the female slim ideal, whereas boys experience teasing and exclusion if they are too thin and do not fit the male muscular ideal. As the majority of studies have found evidence for the first hypothesis, we predicted that overweight girls and boys would report higher levels of all kinds of peer pressure (i.e. teasing, exclusion, influences by friends, pressure from school and class norms).

5. Based on previous studies we further expected that overweight adolescents would experience more *parental teasing* as well as *encouragement to control weight and shape*.

## Method

### Participants and procedure

This study reports on the baseline survey of a longitudinal investigation for which the procedure was approved by the ethics commission and the local ministry of education. The study was conducted among middle- and upper-class students in grades 7 to 9 in six German high schools that cooperate with our institution for different research projects. Teachers delivered written information to the students and their parents and collected informed consent forms from those who agreed to participate. Of the 1,342 students who received information, 1,113 (83%) returned their consent forms and completed the questionnaire during a regular lesson. One case was excluded due to invalid data. Demographic information for the remaining sample of 1,112 students is given in Table 1.

**Table 1:** Demographic Characteristics of the Sample (N = 1,112)

	**Girls (*n *= 603)**		**Boys (*n *= 509)**		***p***
**Age**	10 – 16 years		11 – 16 year		.003
	*M* = 13.32, *SD* = 0.79		*M* = 13.46, *SD* = 0.83		
**Grade**	36.7%	grade 7	33.8%	grade 7	*n.s.*
	36.3%	grade 8	36.9%	grade 8	
	27.0%	grade 9	29.3%	grade 9	
**BMI**	*M* = 18.63, *SD* = 2.57		*M* = 18.70, *SD* = 2.73		*n.s.*

### Measures

#### Weight status

Body-mass index (BMI) was computed based on self-reported age, weight, and height. Self-reported weight is proven to be a valid measure in epidemiological studies with adolescents [36]. The percentile ranking of BMI was assigned using the WHO norms for age and gender [37]. Following Jones and Crawford [7] weight status was classified as follows: underweight (BMI < 25^th^ percentile), low average weight (25^th^ ≤ BMI < 50^th^ percentile), high average weight (50^th^ ≤ BMI < 85^th^ percentile), and overweight (BMI ≥ 85^th^ percentile).

#### Appearance-related social pressure

The assessment of social pressure has been limited in previous research. Studies that explored mechanisms of sociocultural pressure predominantly asked about a general feeling of pressure to be thin often with single items (e.g. [11,14], The Perceived Sociocultural Pressure Scale [38]). Moreover, several studies applied measures to focus on specific aspects of pressure (e.g., peer influence: Inventory of Peer Influence on Eating Concerns (IPIEC [19]); family influence: Family Influence Scale (FIS [39]); and teasing: Perception of Teasing Scale (POTS [40]). Because most of these items imply a connotation towards a thin ideal, they are probably not suitable among boys and might thus lead to underestimations of the relevance of pressure among boys.

Because to our knowledge no instrument exists that measures social pressure from peers and parents simultaneously while distinguishing various types of pressure, we developed the Appearance-Related Social Pressure Questionnaire (FASD, Fragebogen zum aussehensbezogenen sozialen Druck [17]). To gather an accurate measure of social pressure we included on the one hand those social impacts established in the literature and on the other hand conducted qualitative interviews with adolescent girls and boys exploring important sources of social pressure in their daily lives. The literature predominantly provides evidence for comparable risk factors for body concerns in both boys and girls [e.g. [41,42]. The findings from our interviews during the item generation also pointed to comparable forms of social pressure. However, we had to ensure that the phrases were suitable for both girls and boys as well as for adolescents with different weight statuses. Thus, we used general terms like “appearance“ or “body shape“ and tried to avoid specific ones like “thinness“ to avoid a bias. The 32 items are rated on a 5-point Likert scale from 1 (*strongly disagree*) to 5 (*strongly agree*). A series of structural equation models was used to investigate the factor structure of the FASD. The best fitting model revealed two parts (peer and parental pressure), each consisting of four scales that comprise four items, respectively, and ask about different types of appearance-related social pressure.

The section on *parental pressure* comprises four scales:

▌ *Parental Teasing* (α = .83, *r*_*tt*_ = .60): This scale combines direct aspects of pressure from parents such as negative comments or disparaging gestures.

▌ *Injustice and Ignorance* (α = .65, *r*_*tt*_ = .72): By measuring the feeling of only being accepted when looking better or being ignored for not looking good, the second scale implies an indirect kind of pressure. Although we could not find previous literature that directly investigated this parental impact, it was mentioned by the adolescents that were interviewed during the construction of the FASD, and the findings of Meesters et al. [13]. also suggested such aspects of parental pressure.

▌ *Parental Encouragement to Control Weight and Shape* (α = .79, *r*_*tt*_ = .81): The third scale includes also direct – but in contrast to the first scale, not obviously disparaging – comments by parents as it measures parental encouragement to pay heed to one’s body shape.

▌ *Parental Norms and Modeling* (α = .74, *r*_*tt*_ = .83): Finally, the fourth parental scale comprises indirect pressure through parental standards of appearance and efforts to look good.

The section *peer pressure* comprises the following four scales:

▌ *Peer Teasing* (α = .78, *r*_*tt*_ = .83): Comparable to the parental scale, this scale is composed of direct types of pressure like disparaging comments and gestures by peers.

▌ *Exclusion* (α = .81, *r*_*tt*_ = .86): This scale asks about the feeling of being ignored or excluded from social events because of one’s appearance.

▌ *School and Class Norms* (α = .78, *r*_*tt*_ = .69): The third scale measures an indirect aspect of peer pressure as it inquires about the importance of appearance in school and class.

▌ *Modeling by Friends* (α = .73, *r*_*tt*_ = .72): The final peer pressure scale asks about appearance standards of friends and efforts to achieve that standard, which can also be seen as an indirect aspect of peer pressure.

The internal consistency scores were taken from the current sample, whereas the test-retest reliability coefficients were obtained in a previous study. Intercorrelations between the FASD-scales in this study were predominantly moderate (r = .13 to .55). Only teasing by peers and exclusion showed a higher association (r = .68). The FASD has been used in different studies to ensure its psychometric quality [e.g. [17,43]. Reliability was acceptable for all scales and evidence for factorial, convergent, and incremental validity has been determined [17]. Details on the construction and validation of the FASD are available on:

http://www.psych.uni-potsdam.de/counseling/research/messure-e.html.

### Statistical analyses

All statistical analyses were performed using SPSS 15.0. Because missing data rates were below 5% common EM-substitution was applied. We conducted preliminary analyses using ANOVA and the chi square test to investigate the characteristics of the sample and differences in the group formation. In order to investigate differential effects in the perception of different types of social pressure we conducted a 2 (gender) x 3 (grade-level) x 4 (BMI category) multivariate analysis of variance (MANOVA) including the mean scores of all FASD subscales. We decided to include gender, grade-level, and BMI in one analysis, because different authors have discussed interactive effects of gender, weight, and age and, moreover, we wanted to account for confounding effects because our data suggested associations between the factors. Furthermore, MANOVA was chosen due to the substantial intercorrelations among the different FASD scales. Wilks’ Lambda will be reported as the multivariate test criterion. For the post hoc univariate analysis, the significance level was adjusted using Bonferroni correction (*p* < .006).

## Results

### Preliminary analyses

Preliminary analyses revealed that the boys in our sample were slightly older, *t* (1110) = 2,94, *p* <.01, and significantly more of them could be classified as being overweight, χ^2^ (3, n = 1112) = 9.17, *p* < .05 (Table 2). In addition, students in grades 7 to 9 significantly differed as to mean age with only marginal overlaps in range, *F* (2, 1109) = 1237.50, *p* < .001. Hence, the mean age in grade 7 was: *M* = 12.66 (*SD* = 0.43), in grade 8: *M* = 13.33 (*SD* = 0.45) and in grade 9: *M* = 14.36 (*SD* = 0.47). Finally, analyses indicated that BMI significantly increased with age, *F* (2, 1109) = 3.57, *p* <.001. However, no differences could be found regarding the distribution of the four weight status groups according to gender and grade. With the use of MANOVA, we could account for the variations between the groups.

**Table 2:** BMI Groups by Gender and Grade (N = 1,112)

	**Underweight**	**Low average-weight**	**High average-weight**	**Overweight**	***p***
	**(< 25**^**th **^**percentile)**	**(25**^**th **^**≥ BMI < 50**^**th **^**percentile)**	**(50**^**th **^**≥ BMI < 85**^**th **^**percentile)**	**(BMI ≥ 85**^**th **^**percentile)**	
**Girls **	32.7%	27.5%	31.3%	8.5%	.027
**Boys**	27.7%	25.1%	34.0%	13.2%	
**Grade 7**	33.6%	27.2%	27.2%	12.0%	*n.s.*
**Grade 8**	29.5%	27.0%	32.9%	10.6%	
**Grade 9**	27.6%	24.7%	38.8%	9.0%	

*Note.* The BMI ranking was assigned using the WHO norms and weight status was classified following Jones & Crawford (2006).

### Overall results

The overall 2 (gender) x 3 (grade-level) x 4 (BMI category) MANOVA of the different aspects of social pressure did not show a significant overall interaction between gender, grade, and weight status but did reveal main effects for all the three factors. Hence, the MANOVA revealed a significant main effect for gender, *F*(8, 1081) = 16.64, *p* < .001, η^2^ = .11, which was of medium size (Table 3). Furthermore, we found a moderate main effect for grade-level, *F*(16, 2164) = 5.91, *p* < .001, η^2^ = .04 (Table 4) and a moderate main effect for BMI category, *F*(24, 3249) = 7.01, *p* < .001, η^2^ = .05 (Table 5).

**Table 3:** Main effects in appearance-related social pressure for gender

	**Girls**	**Boys**	
	**( *n *=603)**	**( *n *=509)**	
	***M***	***M***	
	***(SD)***	***(SD)***	
**Parental Pressure**			
Parental Teasing	1.18	1.11	η^2^ = .01**
	(0.48)	(0.30)	
Injustice & Ignorance	1.13	1.14	
	(0.33)	(0.30)	
Parental Encouragement	1.65	1.70	
	(0.76)	(0.74)	
Parental Norms & Modeling	2.12	2.12	
	(0.74)	(0.76)	
**Peer Pressure**			
Peer Teasing	1.57	1.49	η^2^ = .01***
	(0.68)	(0.60)	
Exclusion	1.97	1.71	η^2^ = .05***
	(0.82)	(0.69)	
School & Class Norms	2.18	1.99	η^2^ = .03***
	(0.81)	(0.71)	
Modeling by Friends	2.61	2.22	η^2^ = .06***
	(0.76)	(0.73)	

*Note.* The items are rated on a 5-point Likert scale from 1 (*strongly disagree*) to 5 (*strongly agree*).

*p < .05; **p < .01; *** *p* < .001.

**Table 4:** Main effects in appearance-related social pressure for grade

	**Grade 7**	**Grade 8**	**Grade 9**	
	**( *n *=393)**	**( *n *= 407)**	**( *n *=312)**	
	***M***	***M***	***M***	
						***(SD)***	***(SD)***	***(SD)***
**Parental Pressure**				
Parental Teasing	1.10	1.16	1.19	
	(0.34)	(0.45)	(0.44)	
Injustice & Ignorance	1.12	1.14	1.15	
	(0.31)	(0.33)	(0.31)	
Parental Encouragement	1.58^a^	1.73^b^	1.71^b^	η^2^ = .01**
	(0.72)	(0.78)	(0.75)	
Parental Norms & Modeling	1.99	2.15	2.24	
	(0.74)	(0.71)	(0.80)	
**Peer Pressure**				
Peer Teasing	1.44^a^	1.56^b^	1.62^b^	η^2^ = .02***
	(0.60)	(0.64)	(0.68)	
Exclusion	1.73^a^	1.94^b^	1.89^b^	η^2^ = .02***
	(0.70)	(0.83)	(0.77)	
School & Class Norms	1.83^a^	2.14^b^	2.37^c^	η^2^ = .06***
	(0.64)	(0.74)	(0.85)	
Modeling by Friends	2.25^a^	2.49^b^	2.59^b^	η^2^ = .02***
	(0.76)	(0.73)	(0.78)	

*Note.* The items are rated on a 5-point Likert scale from 1 (*strongly disagree*) to 5 (*strongly agree*). Means with the same subscript are not significantly different.

*p < .05; **p < .01; *** *p* < .001.

**Table 5:** Main effects in appearance-related social pressure for BMI - categories

	**Under**	**Low**	**High**	**Over**	
	**( *n *=338)**	**( *n *=294)**	**( *n *=362)**	**( *n *=118)**	
	***M***	***M***	***M***	***M***	
	***(SD)***	***(SD)***	***(SD)***	***(SD)***	
**Parental Pressure**					
Parental Teasing	1.11	1.13	1.18	1.22	
	(0.34)	(0.35)	(0.45)	(0.56)	
Injustice & Ignorance	1.09	1.13	1.18	1.15	
	(0.22)	(0.29)	(0.39)	(0.35)	
Parental Encouragement	1.53^a^	1.59^a^	1.70^a^	2.18^b^	η^2^ = .07***
	(0.66)	(0.68)	(0.77)	(0.89)	
Parental Norms & Modeling	2.01	2.16	2.19	2.17	
	(0.69)	(0.73)	(0.81)	(0.78)	
**Peer Pressure**					
Peer Teasing	1.38^a^	1.43^a^	1.62^b^	1.98^c^	η^2^ = .09***
	(0.49)	(0.50)	(0.68)	(0.92)	
Exclusion	1.68^a^	1.73^a^	1.97^b^	2.29^c^	η^2^ = .08***
	(0.62)	(0.66)	(0.85)	(0.97)	
School & Class Norms	1.98^a^	2.05^ab^	2.22^b^	2.15^ab^	η^2^ = .02**
	(0.70)	(0.74)	(0.83)	(0.81)	
Modeling by Friends	2.33	2.44	2.52	2.45	
	(0.74)	(0.76)	(0.78)	(0.80)	

*Note.* Under = underweight (BMI < 25^th^ percentile), Low = low average (25^th^ ≤ BMI < 50^th^ percentile), High = high average (50^th^ ≤ BMI < 85^th^ percentile), Over = overweight (BMI ≥ 85^th^ percentile). The items are rated on a 5-point Likert scale from 1 (*strongly disagree*) to 5 (*strongly agree*). Means with the same subscript are not significantly different.

*p < .05; **p < .01; *** *p* < .001.

### Main effects for gender

#### Hypothesis 1

With regard to gender effects we expected a main effect indicating that girls display higher levels on all aspects of appearance-related social pressure from peers and parents. However, follow-up univariate tests confirmed the main effect for gender only for one aspect of parental pressure. Hence, girls reported more parental teasing, *F*(1, 1088) = 10.81, *p* < .01, η^2^ = .01, which constitutes a small effect. Furthermore, girls displayed higher scores on all peer pressure scales. More specifically, we found small effects regarding peer teasing, *F*(1, 1088) = 13.11, *p* < .001, η^2^ = .01; exclusion, *F*(1, 1088) = 53.81, *p* < .001, η^2^ = .05; and school and class norms, *F*(1, 1088) = 29.77, *p* < .001, η^2^ = .03 but for modeling by friends the effect is even of medium size, *F*(1, 1088) = 72.58, *p* < .001, η^2^ = .06.

In sum, gender differences in peer pressure were noteworthy and indicated that girls perceived more pressure from peers compared to boys, while the largest difference was revealed for modeling by friends.

### Main effects for grade-level

#### Hypothesis 2

We hypothesized that the impact of friends and schoolmates would be higher in older compared to younger adolescents. In contrast to our hypothesis, differences emerged not only for modeling by friends, *F*(2, 1088) = 12.80, *p* < .01, η^2^ = .02, and school and class norms, *F*(2, 1088) = 35.29, *p* < .001, η^2^ = .06, but also for peer teasing, *F*(2, 1088) = 8.03, *p* < .001, η^2^ = .02 , and exclusion, *F*(2, 1088) = 8.85, *p* < .001, η^2^ = .02. Bonferroni post hoc tests were used to evaluate differences between grade-levels (corrected *p* < .017) and revealed that students from grade 7 reported significantly lower levels on all peer pressure scales compared to students from grades 8 or 9. Only regarding school and class norms could a significant difference be found between students from grades 8 and 9. As reflected by the effect sizes, grade differences for school and class norms were particularly evident.

#### Hypothesis 3

Regarding variations by grade-level we expected that parental encouragement to control weight and shape would be more prevalent among older adolescents. The univariate follow-up analyses of grade differences (corrected *p* < .006) confirmed this hypothesis and a main effect for parental encouragement to control weight and shape revealed, which was perceived to a lesser degree in grade 7 compared to grade 8, *F*(2, 1088) = 6.48, *p* < .01, η^2^ = .01.

### Main effects for body mass

#### Hypothesis 4

Finally, we predicted that overweight adolescents would report higher levels of all types of peer pressure. Univariate tests (corrected *p* < .006) combined with Bonferroni post hoc tests were used to evaluate differences between BMI categories (corrected *p* <.008). With regard to peer pressure, small effects for school and class norms, *F*(3, 1088) = 5.56, *p* < .01, η^2^ = .02, emerged. Interestingly, we found the highest scores among high-average-weight students. Post hoc tests indicated that high-average weight students scored significantly higher than underweight students on school and class norms. Main effects for peer teasing, *F*(3, 1088) = 34.15, *p* < .001, η^2^ = .09 and exclusion, *F*(3, 1088) = 30.28, *p* < .001, η^2^ = .08, proved to be particularly pronounced. Further, there emerged a trend, indicating that the level of peer teasing and exclusion increased with higher weight status. Underweight and low-average weight students displayed the lowest levels and did not differ in their scores. In contrast to our hypothesis the different weight groups did not differ in the perception of modeling by friends.

#### Hypothesis 5

We further expected that overweight adolescents would experience more parental teasing and encouragement to control weight and shape. Regarding parental pressure, the results supported weight-related differences only for one aspect of parental pressure – encouragement to control weight and shape, *F*(3, 1088) = 25.98, *p* < .001, η^2^ = .07 – indicating a large effect. The trend suggested that parental encouragement to control weight and shape increased with higher weight status. However, the scores only significantly differed for overweight students.

To sum up, our analyses revealed main effects for gender, grade-level, and weight status, but no interaction between these factors. With an effect size of η^2^ = .11, gender differences proved to be particularly pronounced. Girls scored higher on all peer pressure scales and showed slightly higher scores on parental teasing. Moderate main effects for grade-level revealed that students from grade 7 differed from students from grades 8 and 9 on the peer pressure scales. Likewise, students from grade 7 showed low levels of parental encouragement to control weight and shape. Finally, main effects for weight status were particularly pronounced for peer teasing and exclusion as well as for parental encouragement to control weight and shape. The findings indicated that particularly high-average and overweight adolescents perceived appearance pressure.

## Discussion

The relevance of appearance-related social pressure as a crucial factor for low self-esteem and depression as well as body dissatisfaction and unhealthy body change efforts has been proven repeatedly [e.g.[44-46]. Up to now, knowledge of gender, weight, and age-related variations in social pressure has either been incomplete or controversial because very few studies have explicitly investigated these aspects together. Moreover, most of the existing studies have permitted only limited conclusions, because they either focused on single aspects of social pressure or were limited in their assessment.

The current study thus contributes to a better understanding of the occurrence of social pressure by explicitly addressing gender, grade-level, and weight variations in a large sample of German adolescent girls and boys. Furthermore, we applied a new measure (FASD), whose psychometric quality and applicability for both girls and boys has been proven before [17,43] and which allows a broad assessment of aspects of appearance pressure from both peers and parents. In doing so, the results may help to identify adolescents who are particularly at risk of suffering from appearance-related social pressure and thus provide concrete advice for preventive approaches.

Following the overall effects of the current study, the findings suggest that social pressure is more prevalent during mid-adolescence compared to early adolescence and girls and adolescents with higher weight are particularly affected. A comparison of the effect sizes indicated that gender differences were particularly pronounced in the current sample.

### Gender variations

Our hypotheses regarding gender differences in peer and parental pressure were only partly supported. While we found the expected gender differences on all peer pressure scales, gender effects were only found for parental teasing. Thus, our results support previous findings on negative verbal commentary that found a higher prevalence among girls [6,14]. Nevertheless, the conclusion often drawn in previous research that the parental impact is generally higher for girls was probably premature. Because the effect size for parental teasing was rather low and no effects emerged on the other scales, levels of parental pressure among girls and boys seem to be more similar than previously assumed. This finding also resembles the results of Rodgers et al. [24]. Even though they found gender differences for a few aspects of parental pressure, a closer look at the scores and effect sizes reveals that only the difference regarding negative maternal comments is noteworthy. Maybe no effects were revealed because of the extreme floor effect and the restricted variance of these FASD scales, which is also known for instruments assessing similar constructs in population-based samples [6].

Gender effects for peer pressure are in line with current research, indicating that girls are more strongly affected by peer influences and the impact of friends is especially important [7,46]. Gender effects with regard to teasing experiences have been controversial because of limitations in the measurement of teasing. Our results obtained with a gender-neutral, reliable peer teasing scale support the findings of the American EAT-Project [26] and can serve as further evidence that girls experience more peer teasing. Summing up, the results support the assumption that girls are particularly embedded in an appearance culture [1,46]. In detail, the findings suggest that girls perceive more pressure from appearance norms and modeling and are more often subject to proximate forms of peer pressure such as teasing or exclusion. Because the current study applied a measure of social pressure that is not biased by female ideals and has proven to be suitable for both girls and boys alike, we conclude that the higher extent of appearance pressure among females is not just a result of inappropriate measurement but in fact a result of the greater societal emphasis on beauty and appearance for females [5].

### Grade-level variations

The prevalence of appearance-related social pressure especially by peers underlies age-related trends whereas grade-level effects in parental pressure only emerged for encouragement to control weight and shape and were also rather minor.

In contrast to Chen and Jackson [31], these effects proved to be comparable for girls and boys in this German sample. Based on previous results, comparing male body image in Western and Asian cultures, we assume that the divergent results might point to a cultural difference. As Yang, Grey, and Pope [47] revealed, Asian males were less preoccupied with body image than Western males and they discussed interesting reasons why in Western cultures more emphasis is placed on male appearance (e.g. media exposure, decline in traditional male roles).

In accordance with the literature [7,32], differences in our sample were particularly evident comparing early (grade 7) and middle adolescents (grades 8 and 9). Although conclusions must be drawn cautiously due to the cross-sectional design, it seems as if the transition from grade 7 to grade 8 is particularly relevant. Interestingly, differences were mainly reflected by an increase of perceived school and class norms. This effect might be a result of the local school system. In this region of Germany, students change schools between grades 6 and 7. Hence, the adolescents in grade 7 have just joined a new school context and their new schoolmates. This new school context constitutes an important developmental transition, which is associated with changes in social roles and a substantial reorganization of attitudes and beliefs and has been considered a period of risk for problematic behavior [48]. Thus, we believe that among grade 7 students the establishment of norms and group processes has presumably just started. Consequently, we assume that the results probably reflect both – on the one hand, individual changes and transitions throughout adolescence, and on the other hand, the development of the class as a group of shared attitudes and values. It might be an interesting issue for future studies to distinguish between these two processes and figure out which role age per se or the attainment of a certain grade-level plays in this issue. Furthermore, the findings suggest that early adolescence as well as school transitions are crucial periods for establishing prevention programs that counteracts the development of an appearance culture within a class. Again, we have to emphasize that the findings can only lead to cautious conclusions because of the cross-sectional design of the study. Longitudinal studies are needed to confirm these findings.

### Body mass variations

Our results suggest that mainly high-average and overweight adolescents experience more appearance pressure from peers and parents, whereas teasing and exclusion are particularly prevalent. We could not replicate the interaction of weight and gender reported by Jones and Crawford [7], who hypothesized that girls experience teasing for higher weight whereas boys are teased for being underweight. In line with stigmatization research [49] and our expectations, the results suggest that overweight adolescents are generally faced with more appearance pressure regardless of their gender. A possible explanation for this is methodological, for we could also find slight similar tendencies in the univariate but not in the multivariate analysis. Jones and Crawford [7] also used univariate analyses. However, due to the intercorrelations between the aspects of pressure, we decided to use a multivariate and thus, more conservative approach, which reveals that the interactive effects are not strong enough and that only the main effect of BMI is relevant. Thus, our findings indicate that girls and boys with higher weight are equally at risk of being faced with appearance pressure.

Body mass variations in the perception of more subtle, norm-related aspects of pressure have rarely been investigated and could only be observed to a lesser extent in our sample. However, small effects for school and class norms indicated that high-average students show the highest levels. Possibly, adolescents who barely fail to fit the slim norm are more likely to internalize appearance ideals [46] and are thus more sensible to subtle appearance-related messages.

Regarding parental pressure the body mass effect is primarily reflected in higher levels of parental encouragement to control weight and shape especially among overweight participants. Hence, overweight adolescents perceive their parents as more demanding regarding weight or shape control. This result is not surprising, because parents are often concerned about the overweight of their child and feel responsible [50]. So, they probably try to support weight control and dieting efforts with comments designed to act as reminders. In accordance with previous studies [13,15,25] our findings can serve as further evidence that these encouraging messages are more problematic than previously assumed. The results indicate that the line is fine between support and pressure and future research must keep track of possible consequences. Beyond this, the findings appear to be particularly relevant for the field of obesity prevention and treatment of children and adolescents. Approaches including parents should address these processes and negotiate the balancing act in teaching parents to support their children without putting them under pressure.

The results of our study are limited to a certain extent first due to the sample. Unfortunately, we could not use the data collected on SES and ethnicity of the sample, because plausibility checks revealed that some adolescents misunderstood these items. Hence, we had to consult data from the Federal Statistical Office, which show that Potsdam is a city with a low percentage of inhabitants with foreign backgrounds and a high percentage of inhabitants with academic and higher social background. Because we only included students from schools with higher educational levels generalizations are restricted and future research might extend these findings to larger, more representative samples. Furthermore, the use of self-reported weight remains a limitation when investigating body mass variations. However, self-reported weight has repeatedly proven to be a valid measure in epidemiological studies with adolescents [12,36]. In addition, BMI confounds lean mass with fat mass, which might lead to a screwed picture when studying males. Therefore future research should also include fat-free mass, body fat indices or girth measurements in order to confirm these findings. Finally, the results are based on cross-sectional data and thus do not permit developmental conclusions. The age-related variations can only point to possible trends that require further confirmation in longitudinal studies.

## Conclusion

By investigating a broad range of aspects of social pressure in a large sample of adolescent girls and boys, the current study points to interesting issues regarding age-, gender-, and weight-based risks for appearance-related social pressure. On this basis the results of the current study could enhance the current state of theory on appearance-related social pressure and pointed out the following:

a) Girls in general are not more affected by social pressure. Differences in parental pressure seem negligible. However, gender variations regarding peer pressure are noteworthy.

b) Older students experience more peer pressure. The crucial moment seems to be the transition from grade 7 to grade 8. Age-related variation in parental pressure did not notably occur.

c) Higher weight is associated with higher levels of proximate individual-related appearance pressure (e.g. teasing, exclusion, and parental encouragement), while effects regarding norm-related forms of pressure were rather small. An interaction of weight and gender could not be replicated.

The findings provide suggestions for preventive efforts. Approaches are needed that strengthen those adolescents who are particularly at risk - in our study, these were girls and adolescents with higher weight status. At the same time the results point to the relevance of peers in the exertion of appearance pressure. Hence preventive approaches should bring up the topic of appearance pressure in a school-based context, since early adolescence and school transition appear to be crucial periods for these efforts.

## Competing interests

The authors declare they have no competing interests.

## Authors’ contribution

SH conceived the study, participated in the design and data collection, performed the statistical analyses and drafted the manuscript. PW designed the project in which the study was conducted, obtained funding, participated in its design and coordination and supervised the data analyses and the writing process. All authors read and approved the final manuscript.
